# Modulation of CXC Chemokine Receptor Expression and Function in Human Neutrophils during Aging In Vitro Suggests a Role in Their Clearance from Circulation

**DOI:** 10.1155/2009/790174

**Published:** 2009-04-15

**Authors:** Katja C. Weisel, Frank Bautz, Gabriele Seitz, Sedat Yildirim, Lothar Kanz, Robert Möhle

**Affiliations:** Department of Medicine II, University of Tübingen, Otfried-Müller-Street 10, 72076 Tübingen, Germany

## Abstract

In mice, differential regulation of CXC chemokine receptor expression in circulating polymorphonuclear neutrophils (PMNs) undergoing senescence results in homing to the bone marrow. However, the role of this compartment and of the chemokine receptor CXCR4 is still under discussion, and only scarce data exist about CXCR4 function in human PMN. In our study, we provide evidence that also in human neutrophils, expression (cell surface and mRNA), chemotactic and signaling functions of the homing-related chemokine receptor CXCR4 are upregulated during aging in vitro, independent of addition of stimulatory cytokines (TNF, IL-1, IL-8, G-CSF). In contrast, interleukin-8 receptors are downmodulated (CXCR2) or remain unchanged (CXCR1), suggesting that human PMNs undergoing senescence acquire a phenotype that impairs inflammatory extravasation and favors homing to the bone marrow or other tissues involved in sequestration. Partially retained responsiveness to interleukin-8 may be important for neutrophil function when senescence occurs after extravasation in inflamed tissues.

## 1. Introduction

After release from the bone marrow, mature neutrophils (polymorphonuclear neutrophils (PMNs)) are either rapidly recruited to sites of inflammation, where they represent the immediate defense against bacterial and fungal microorganisms [1, 2], or undergo senescense within hours, which results in apoptosis and rapid clearance from the circulation [3]. Without efficient removal of aged neutrophils, the release of reactive oxygen species, proteases, and other cytotoxic enzymes from the granules of PMN during uncontrolled apoptosis and degradation would result in tissue damage [4]. PMNs that enter inflammatory sites have a prolonged life-span that allows efficient phagocytosis of bacteria before they become apoptotic and are ultimately eliminated by tissue macrophages [5]. By which mechanism senescent neutrophils are cleared from the circulation in vivo is not completely understood. It is believed that aged PMNs acquire functional defects [6] and are subsequently recognized and phagocytosed by macrophages [7]. However, for a rapid and efficient clearance, additional mechanisms must exist to selectively direct senescent neutrophils from the bloodstream into the tissue where marcophages reside, which is reminiscent of lymphocyte or hematopoietic stem cell homing.

The chemokine receptor CXCR4 and its ligand CXCL12 (stromal cell-derived factor-1, (SDF-1)) have emerged as major factors regulating bone marrow homing of circulating hematopoietic stem cells, either mobilized endogenously after cytotoxic damage or infused therapeutically during stem cell transplantation [8]. In both instances, stem cell homing occurs rapidly within hours, which implies that a similar chemokine receptor-based homing-like mechanism could also contribute to neutrophil clearance from the circulation. However, in contrast to hematopoietic stem cells that intrinsingly express high levels of CXCR4, the expression level of this chemokine receptor is low in neutrophils [9]. Indeed, differential regulation of CXCR4 and of the interleukin-8 (IL-8) receptor CXCR2 appears to control the release of murine neutrophils and their return to the bone marrow after senescence, as aging is associated with upregulation of CXCR4 and loss of CXCR2 on the cell surface [10]. These and other studies also showed that senescence of neutrophils, a process which is almost solely dependent on time and temperature, takes place not only in vivo, but also during culturing without stimulatory cytokines in vitro [11]. However, regulatory mechanisms observed in murine neutrophils that involve IL-8-mediated effects may be different in the human system. For instance, while human neutrophils express two IL-8 receptors (CXCR1 and CXCR2), [12] only the CXCR2 homologue is found in mice.

In contrast, other in vitro and in vivo studies rather suggested that downregulation of CXCR4 occured during maturation of neutrophils, which was further augmented by activation [13–15]. However, neutrophils recovered from sites of inflammation as analyzed by Suratt et al. [13, 14] are different from aged PMN in the bloodstream. Thus, the idea that upregulation of CXCR4 is a key mechanism involved in the clearance of aged neutrophils is not generally accepted. We therefore analyzed cell surface and mRNA expression as well as function of CXCR4 in human granulocytes undergoing aging in vitro. Our results support the concept that upregulation of CXCR4 in senescent granulocytes is an active process, which results in increased responsiveness to SDF-1, and supports their clearance from the circulation in a homing-like fashion.

## 2. Materials and Methods

### 2.1. Cell Lines, Isolation of Neutrophils, and Aging In Vitro

The myeloid cell lines KG1a and HL-60 were cultured in IMDM or RPMI 1640 medium (Seromed-Biochrom) supplemented with 10% FCS. Peripheral blood (PB) was obtained from normal volunteers after informed consent according to the Ethical Committee of the University of Tübingen (Project no. 268/2003). Isolation of mononuclear cells (MNCs) and polymorphonuclear neutrophils (PMNs) was performed using dextran sedimentation, Ficoll density centrifugation, and NH_4_Cl lysis as described previously [16]. PMNs (purity >98%) were resuspended in serum-free medium (X-Vivo 20, BioWhittaker, Walkersville, Md, USA), and incubated at 37°C/5% CO_2_ in 6-well plates for up to 18 hours to allow aging [10]. In some experiments, stimulatory cytokines were added: rhG-CSF (recombinant human granulocyte-colony-stimulating factor) at 100 ng/mL (Amgen, Thousand Oaks, Calif, USA), rhTNF-alpha (recombinant human tumor-necrosis factor-alpha) at 20 ng/mL, IL-1-beta (recombinant human interleukin-1-beta) at 20 ng/mL, and rhIL-8 (recombinant human interleukin-8), at 100 ng/mL (PeproTech Inc., New York, NY, USA). Following incubation all cells were removed from the flask (adherent cells were removed using 0.2 g/L EDTA in balanced saline) and used for flow cytometric analysis. As a control, granulocytes were kept at 4°C. 

### 2.2. Flow Cytometry

Expression of CXCR1, CXCR2, and CXCR4 on PMN was analyzed by flow cytometry as described previously [17]. The mean fluorescence intensity was calculated from the fluorescence histograms and expressed in arbitrary units.

### 2.3. Northern Blot Analysis

Total RNA was isolated using RNeasy Mini Kit (Qiagen, Hilden, Germany), and 1-2 *μ*g were separated in a 1% agarose formaldehyde gel and transferred onto positively charged nylon membranes by capillary transfer. A 484 bp DNA probe for detection of CXCR4 mRNA was amplified by PCR from CXCR4 cDNA as described previously [17]. As control, a 513 bp beta-actin probe was generated by PCR amplification. DIG-labeling of the PCR-products and the colorimetric detection was performed using the “DIG DNA Labeling and Detection Kit” (Boehringer Mannheim, Mannheim, Germany).

### 2.4. Western Blot Analysis

Freshly isolated PMNs were cultured in X-VIVO20 medium for 18 hours at 37°C or kept at 4°C, respectively. After stimulation with 100 ng/mL SDF-1 for the indicated periods of time at 37°C, whole cell lysates were prepared by resuspending the pelleted cells in buffer containing 50 mM HEPES (pH 7.5), 10% glycerol, 1% Triton X-100, 1.5 mM MgCl_2_, 150 mM NaCl, protease inhibitors (Complete Mini; Roche, Mannheim, Germany) and the phosphatase inhibitors NaF (100 mM), Na_4_P_2_O_7_ (10 mM) and activated Na_3_VO_4_ (1 mM; all from Sigma). Equal amounts of protein were separated on a 10% SDS-polyacrylamide gel, and transferred onto nitrocellulose membrane (Schleicher & Schuell, Dassel, Germany). The blots were probed with phospho-specific polyclonal antibodies against p44/42 MAP Kinase (Thr202/Tyr204), or with the control antibody against the nonphosphorylated form (Cell Signaling Techn., Beverly, Mass, USA). Bands were visualized by ECL staining (Amersham Biosciences, Freiburg, Germany) and analyzed by densitometry using the Scion Image for Windows software (Scion Corporation; Frederick, Md, USA). The value obtained from the phospho-MAPK band was divided by the value of the corresponding total MAPK band for each sample (relative phospho-MAPK intensity). For all experiments, identical reagents, concentrations and exposure times were used.

### 2.5. Transendothelial Migration

In vitro analysis of migration across microvascular endothelium was performed as described previously [18], using the human endothelial cell line BMEC-1 grown on 3 *μ*m transwell microporous membranes (Transwell, Corning-Costar, Bodenheim, Germany). rhSDF-1alpha (recombinant human stromal cell-derived factor-1, R&D Systems, Wiesbaden, Germany) and rhIL-8 (recombinant human interleukin-8, PeproTech Inc., New York, NY, USA) were added to the lower chamber (underneath the Transwell inserts) at 100 ng/mL. Blocking CXCR4 mAb (clone 12G5, Pharmingen) was added to the cells in the upper chamber at a final concentration of 10 *μ*g/mL. Transmigrated cells were enumerated after 3 hours.

### 2.6. Statistical Analysis

Results are expressed as the mean ± standard error of the mean (SEM) from at least 3 independent experiments. Analysis of data was performed using SigmaStat 2.03 software (SPSS Inc.). Differences between groups were compared by Student's *t*-test (e.g., chemokine-induced migration of freshly isolated PMN versus control) or, where applicable (chemokine receptor expression 0 hours versus 18 hours and migration of aged neutrophils versus cells kept at 4°C), paired t-test, and considered significant at *P* ≤ .05.

## 3. Results

### 3.1. Aging of Human Neutrophils In Vitro Resulted in Increased Cell Surface and mRNA Expression of CXCR4, but Not CXCR1 and CXCR2

**Figure 1:**
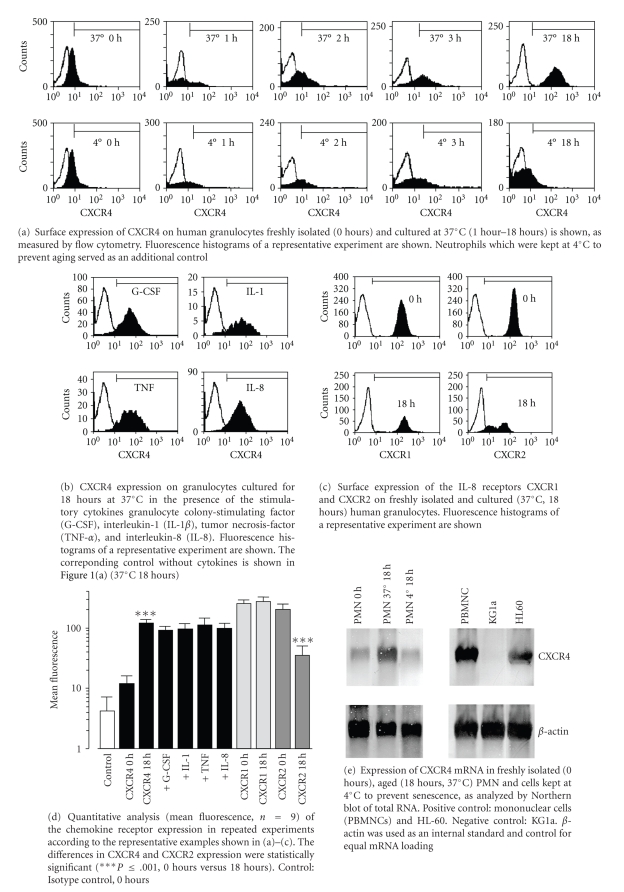
Expression of CXCR4, CXCR1, and CXCR2 in human granulocytes during aging in vitro.

By flow cytometry, cell surface expression of CXCR4 on human PMN was very weak immediately after isolation from peripheral blood as shown in Figures 1(a) and 1(d). Aging for up to 18 hours in vitro (37°C/5% CO_2_) resulted in a significant upregulation of CXCR4 on the cell surface, as reflected by a shift of the mean fluorescence of at least one log step. Increased CXCR4 expression was detected starting from 3 hours and reached maximal levels after 12–18 hours. As an additional control, PMNs from the same blood sample were kept at 4°C to inhibit aging. In these cells, an increase in CXCR4 expression was not detected, indicating that upregulation of CXCR4 with age is an active process depending on an intact cell metabolism (Figures 1(a) and 1(d)). We also incubated freshly isolated human PMN with a panel of stimulatory cytokines for 18 hours. Addition of G-CSF, IL-1, TNF, or IL-8 to the cells did not influence the observed increase of CXCR4 on the cell surface (Figures 1(b) and 1(d)). 

As IL-8/CXCR2 were proposed to be involved in murine neutrophil homeostasis [10], we included expression analysis of the two human CXC receptors recognizing IL-8 (CXCR1 and CXCR2) in our experiments. In contrast to CXCR4, expression of both receptors was relatively high in freshly isolated cells with unchanged CXCR1 expression during aging for 18 hours, whereas CXCR2 was even significantly reduced on the cell surface, as shown in Figures 1(c) and 1(d). As changes of the cell surface expression of chemokine receptors may depend on posttranslational effects such as receptor internalisation and recycling, we used Northern blot for quantitative mRNA analysis and could demonstrate increased CXCR4 mRNA expression in PMN aged in vitro (18 hours) compared to freshly isolated cells, as shown in Figure 1(e). These results indicate that both increased mRNA transcription and consecutive protein synthesis contribute to upregulation of CXCR4 expression on the cell surface in PMN undergoing senescence rather than only re-expression of stored and/or recycled CXCR4 receptor.

### 3.2. Increased SDF-1-Induced Migration Was Observed in Aged Neutrophils

**Figure 2:**
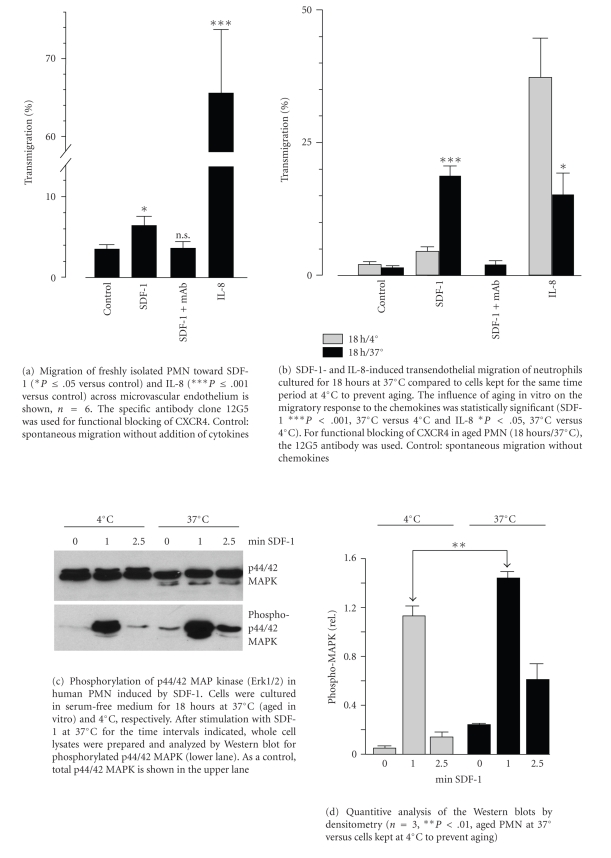
Effects of SDF-1 and IL-8 on transendothelial migration and p44/42 MAP kinase phosphorylation in human PMN in vitro.

As shown in Figure 2(a), transendothelial migration of freshly isolated PMN toward SDF-1 in vitro was approximately twofold increased compared to spontaneous migration and statistically significant. The migratory effect of SDF-1 was completely blocked by the CXCR4 monoclonal antibody 12G5, indicating that migration toward SDF-1 was exclusively mediated by CXCR4. In accordance with the observed high expression of CXCR1 and CXCR2 in freshly prepared PMN, migration toward IL-8 was markedly greater than both spontaneous and SDF-1-mediated migration. Furthermore, we were interested to know if the observed changes in CXCR4 and CXCR2 surface expression during aging (Figure 1(d)) were reflected in functional tests. Indeed, migration toward SDF-1 was much more pronounced in aged PMN (about tenfold increase compared to spontaneous migration, Figure 2(b)) than in freshly isolated PMN (twofold increase, Figure 2(a)). SDF-1-induced migration of aged PMN was reduced to the baseline level in the presence of the blocking CXCR4 antibody. In contrast to PMN undergoing senescence, SDF-1-induced migration of cells kept at 4°C for the same time period was only marginally increased (Figure 2(b)). However, the migratory response of aged PMN to IL-8 was significantly reduced after aging compared to cells kept at 4°C, but still considerably greater than spontanous migration. Indeed, IL-8-induced transendothelial migration of aged neutrophils in vitro was even slightly smaller than that induced by SDF-1.

### 3.3. p44/42 MAP Kinase Signaling following Stimulation with SDF-1 Was Enhanced in Aged Neutrophils

Without stimulation, only weak basal levels of phosphorylated p44/42 MAP-kinase/Erk1/2 were detected in aged PMN and cells kept at 4°C by Western blot analysis (Figure 2(c)). However, in both conditions, strong phosphorylation was observed within one minute after stimulation with 100 ng/mL SDF-1. Phosphorylation of cells kept at 4°C was transient and returned to the baseline level after 2.5 minutes. The response was significantly stronger (Figure 2(d)) and somewhat more sustained (still detectable at 2.5 minutes) in senescent neutrophils as compared to cells which were kept at 4°C to prevent aging.

## 4. Discussion

In this study, we observed basal levels of the chemokine receptor CXCR4 on the cell surface of native human peripheral blood neutrophils as well as basal mRNA expression, which were associated with a weak, but significant migratory response to SDF-1. This is in contrast to previous studies that could not detect CXCR4 in PMN, neither any effect of SDF-1, [9] but may depend on the sensitivity of the methods employed. After aging in vitro, both an increased mRNA and surface expression of CXCR4 were found, which was associated with more efficient migration across bone marrow endothelium and increased phosphorylation of p44/42 MAP kinase (Erk1/2) in response to SDF-1. In postmitotic PMN, Erk1/2 signaling is not related to cell proliferation, but rather plays a role in the regulation chemotaxis [19, 20]. This is supported by our finding that the MAP kinase phosphorylation in response to SDF-1 was only transient and returned to baseline levels after a few minutes. The effects observed were not due to selection of a subpopulation with increased CXCR4 expression during culturing in vitro, as neutrophils with a similar high CXCR4 level were virtually absent at the beginning of the culture period.

In contrast to a previous study by Martin et al. [10] who analyzed trafficking of aged PMN in vivo in a mouse model, our results indicate that upregulation of CXCR4 during aging in human PMN is not solely due to translocation of intracellular CXCR4 to the cell surface, but also to new receptor synthesis. Previously it has been thought that senescence of neutrophils is associated with a general loss of cellular functions [6, 21]. However, our results confirm the notion that human PMN undergoing senescence can upregulate specific mRNA transcription and even acquire functions such as SDF-1/CXCR4-mediated chemotaxis and signaling. The effect of SDF-1 was specifically mediated by CXCR4, since it was abrogated by a functionally blocking CXCR4 antibody. Aging of PMN is associated with controlled apoptosis, as suggested by Weinmann et al. [22]. According to the results of this study, factors that mediate neutrophil migration such as adhesion molecules are also involved in the regulation of apoptosis. It is therefore conceivable that increased expression of CXCR4 in senescent PMN represents a further event in the regulatory network that controls their apoptosis and neutrophil clearance from the circulation.

Taken together, our results demonstrate that during aging, human PMNs acquire a phenotype that supports migration to areas of high SDF-1 concentration. Although particularly bone marrow homing of both hematopoietic stem cells [23] and murine granulocytes [10] has been shown to depend on SDF-1/CXCR4, production of SDF-1 is by no means confined to the bone marrow. For instance, in acute lymphoblastic leukemia, high expression of CXCR4 generally correlates with the ability of the leukemic cells to infiltrate extramedullary sites [24]. Therefore, SDF-1/CXCR4 could also be involved in PMN sequestration in organs such as the liver and spleen. Other mechanisms like adhesion molecules may additionally determine to which extent the clearance of senescent PMN takes place in the liver, spleen, or bone marrow. Indeed, the adhesion molecule P-selectin expressed in hepatic sinusoids supported phagocytosis of PMN by Kupffer cells in an animal model of inflammation caused by endotoxinemia [25]. Interestingly, vascular selectins are also involved in hematopoietic stem cell homing, which is not solely dependent on SDF-1/CXCR4 [26]. Thus, increased expression and function of CXCR4 is only one of many factors that ensure targeted direction of senecent PMN to their individual site of sequestration. In the liver and in the spleen, macrophages appear to play a more direct role in the clearance of neutrophils [27]. This might be explained by the specific microenvironment, particularly by the open microcirculation of the spleen. In the bone marrow, transendothelial migration and extravasation must have occurred before PMN are phagocytosed by macrophages in the tissue. 

SDF-1 not only may be important as a chemoattractant, but also may directly contribute to the apoptosis of senescent granulocytes, as suggested by Lum et al. [28]. In this study, engagement of CXCR4 in granulocytes resulted in the increase of TNF-related apoptosis-inducing ligand (TRAIL) and TRAIL receptors, which eventually supported apoptosis of these cells. From this study, it can be concluded that once senescent PMNs have reached their site of sequestration, high concentration of SDF-1 released by stromal cells may support the process of apoptosis, which then allows nearby macrophages to immediately phagocytose these cells.

High initial expression of the IL-8 receptor CXCR2 followed by downmodulation during aging also resembles observations in mice [10], and may indicate that CXCR2 is similarly involved in the release of human neutrophils from the bone marrow. Furthermore, CXCR2 is mainly responsible for the IL-8-induced inflammatory neutrophil attachment and chemotaxis, [29], suggesting that the loss of CXCR2 in circulating senescent neutophils also results in impaired recruitment to sites of inflammation. In contrast, expression of CXCR1 remained unchanged which may be important for neutrophils after extravasation. This idea is supported by the previous suggestion that CXCR1 expressed on human neutrophils may require higher concentrations of IL-8 for chemotaxis and, therefore, may be involved in neutrophil activities closer to the site of injury and not in the early attachment and extravasation events [30]. In addition, also SDF-1 is produced at sites of tissue damage, which is mediated by upregulation of the hypoxia-inducible factor-1 (HIF-1) [31]. High expression of CXCR4 could therefore contribute to the attraction of PMN to inflammatory areas after extravasation, even when the process of aging has already been initiated.

**Figure 3:**
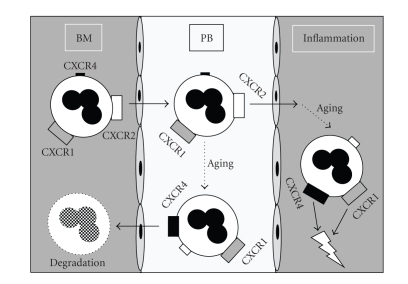
Potential function of CXC chemokine receptors in human neutrophils during aging in vivo. In this model of the putative function of CXC chemokine receptors in aging human neutrophils, the increased expression of CXCR4 in senescent cells circulating in the peripheral blood (PB) is paralleled by loss of CXCR2, resulting in migration to sites with a high SDF-1 concentration such as the bone marrow (BM) or alternatively to other sites of neutrophil sequestration, where they undergo degradation. Similar to in vivo observations in the mouse, CXCR2 might also contribute the release of human neutrophils from the bone marrow. Both IL-8 receptors, CXCR1 and particularly CXCR2, may contribute to extravasation of PMN. In the tissue, further migration toward areas of inflammation (inflamm.) is still mediated by CXCR1 together with CXCR4, even when the process of aging has already started.

Based on our findings and the previous study in mice [10], we propose a model of CXC chemokine receptor modulation during aging (Figure 3) that allows circulating, aged granulocytes to home to the bone marrow (or other sites of sequestration), dependent on CXCR4 and its ligand SDF-1, while after extravasation CXCR1 and CXCR4 sustain attraction of neutrophils to areas of inflammation and tissue damage, where IL-8 and SDF-1 are released.
